# Effect of Laser Quenching-Shock Peening Strengthening on the Microstructure and Mechanical Properties of Cr12MoV Steel

**DOI:** 10.3390/ma15196693

**Published:** 2022-09-27

**Authors:** Aixin Feng, Jian Zhao, Jinhao Lin, Xiaoming Pan, Huibin Feng, Changyu Wang, Zhengyuan Lu

**Affiliations:** 1 College of Mechanical and Electrical Engineering, Wenzhou University, Wenzhou 325035, China; 2 Zhejiang Provincial Key Laboratory of Laser Processing Robots, Machinery Industry Key Laboratory of Laser Processing and Testing, Wenzhou 325035, China; 3 Rui’an Graduate College, Wenzhou University, Wenzhou 325206, China; 4 Zhejiang Linuo Fluid Control Technology Co., Ltd., Wenzhou 325200, China

**Keywords:** laser quenching, laser shock peening, Cr12MoV steel, wear resistance

## Abstract

The automobile covering parts mold is a key piece of equipment in the automobile industry, and its drawbead is the core element that affects the life of the mold and the quality of the parts made. Due to the complex structure of the mold cavity for covering parts, there exist differences between material flow characteristics, load conditions, stress strain, failure forms and so on in the surface of different parts of its drawbead and the different directions of the same part of the drawbead, thus putting forward new requirements for material strengthening. For the differentiated lose efficacy forms of the dangerous end faces of the tension bars, this study carried out research into the effect of laser quenching–shock peening strengthening (LQ-LSP) on the organization, plastic deformation resistance and wear resistance of Cr12MoV steel. It was shown that the microhardness (722.30 HV) and residual stress (−383.84 MPa) of the specimens were further enhanced after laser quenching–shock peening composite strengthening. The residual austenite content of the specimen was reduced to 0.8%, and the eutectic carbide distribution morphology was improved. After three rounds of laser composite peening, the specimens had the smallest displacement of the nanoindentation load–depth curve, which exhibited the greatest nanohardness (20.0 Pa) and modulus of elasticity (565.25 Pa), while reducing the coefficient of friction (0.61) and surface roughness (0.152 Ra). The smooth and flat surface of the specimen with shallow and narrow plow grooves improved the resistance of Cr12MoV steel to plastic deformation and wear.

## 1. Introduction

The automobile covering parts mold is a key piece of equipment in the automobile industry, and its drawbead is the core element that affects the life of the mold and the quality of the parts made. Due to the complex structure of the mold cavity of covering parts, there exist differences between material flow characteristics, load conditions, stress strain, failure forms, and so on at the surface of different parts of its drawbead and the different directions of the same part of the drawbead, thus putting forward new material strengthening needs. In addition, more than 50% of China’s high-end precision automotive molds rely on imports, and the stability, precision, and iteration speed of China’s molds are not excellent compared with the advanced level of foreign molds. In particular, the comprehensive performance of the covering parts mold drawbead and its strengthening process are seriously lagging behind, which has become a key bottleneck in the research and development of automobile cover mold manufacturing. Therefore, there is an urgent need to improve the surface performance of the drawing tendons through surface strengthening technology and thereby improve the service life of automobile covering parts molds [1,2,3,4,5].

Laser quenching (LQ) is a surface heat treatment process commonly used in the tooling industry to improve surface hardness and wear resistance by inducing the generation of a laser-hardened layer [6]. Compared with such traditional quenching processes as flame quenching, LQ, with the advantages of high heating and cooling speed, uniform thickness of the hardened layer, controllable heating depth and heating trajectory as well as small amount of deformation of the workpiece, and so forth, is suitable for the high-precision requirements of the surface treatment of parts [7]. Liu Yun [8] investigated the effect of different laser quenching powers on C12MoV steel, and it was shown that the martensite content and quenched layer thickness of the material increased with increasing laser power but that the hardness of the quenched layer tended to increase and then decrease. Lianghua Shi [9] studied the effects of laser quenching process parameters on the width of the melting unit of high-carbon alloy steel Cr12, and the results showed that the width of the melting unit varies with the laser process parameters, with the largest effect on the width of the unit being the amount of defocusing, followed by frequency, current, and pulse width in order of influence.

Compared to traditional material modification techniques such as extrusion, shot peening and tumbling, laser shock peening (LSP) is a high-intensity (109 W magnitude) and short-pulse (tens of nanoseconds) process that induces large residual surface compressive stresses. The laser acts on the absorber layer through the confining layer, and an ion explosion occurs to generate pressure pulses that propagate to the surface and generate residual compressive stresses capable of obtaining a deeper reinforced impact layer (1 mm) [10]. Therefore, it can generate high residual compressive stress and improve the morphology and distribution of the microstructure, thus effectively improving the hardness, plastic deformation resistance, wear resistance, fatigue strength, and other properties of the material.

At present, there are few studies on the LSP of steel mold surfaces, and mostly they focus on improving the wear and fatigue resistance of aerospace materials such as titanium alloys and aluminum alloys [11,12,13,14]. Hepeng Zhang [15] studied the effect of LSP on the microstructure gradient of TC titanium alloy and proposed a set of predictive life integration methods for the use of laser strengthening technology. Xiaoan Hu [16], using LSP on a TA14 titanium-based alloy, welded joints for surface strengthening, and the study showed that the microhardness of the surface impacted by the laser was significantly improved, and the relocation of fatigue cracks from the high stress concentration to the inner material greatly improved the fatigue resistance and wear resistance of the material. Ranjith Kumar G. et al. [17] investigated the effect of LSP parameters on the surface corrosion properties of Ti6Al4V specimens, and the results showed that the 1064 nm wavelength induced an increase in hardness and generated sufficient compressive stress at a depth of 50 μm at 3 GW/cm^2^. The 532 nm wavelength-reinforced specimens resisted corrosion better than 1064 nm wavelength-reinforced specimens.

At present, with the continuous development of laser processing technology and the increasing requirements for material properties, the laser composite processing process has become a hot spot for current research [18]. The effect of LSP in improving hardness is stronger than the conventional methods of hardening and will form a large residual compressive stress to effectively inhibit fatigue cracking and crack expansion, improve the hardness of the material, refine the tissue grain, and improve wear resistance [19]. Therefore, based on the theoretical basis of LQ and LSP, this paper conducts a study on the local LQ-LSP process of Cr12MoV, exploring the influence of LQ-LSP on the organization and properties of Cr12MoV steel.

## 2. Materials and Methods

This study is about the effect of LQ-LSP on the properties of Cr12MoV steel such as hardness, residual stress, and frictional wear. The test procedure is divided into strengthening test and testing test. The strengthening tests include LQ and LQ-LSP, while the testing tests include surface quality observation, micro-hardness testing, surface residual stress testing, micro-metallographic organization testing, SEM, XRD, and plastic deformation resistance and wear resistance testing.

### 2.1. Materials and Sample Preparation

The experimental specimen is a Cr12MoV steel plate supplied by Taiyuan Iron and Steel Group with the dimensions of 25 mm × 15 mm × 6 mm. Table 1 shows the chemical composition of Cr12MoV steel. Before the experiment, the surface of the sample was polished with diamond sandpaper and then ultrasonically cleaned with ethanol, dried, prepared for use, and then subjected to the LQ-LSP test.

### 2.2. Experimental Equipment

**Figure 1:**
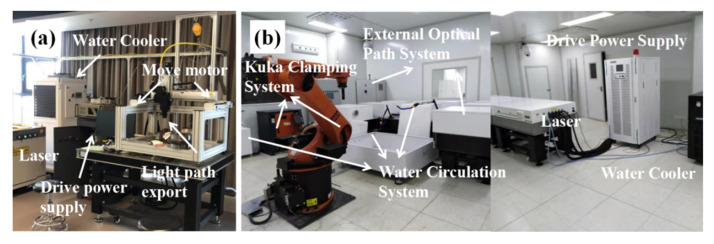
Surface strengthening equipment—(**a**) laser-processing drive control all-in-one system; (**b**) equipment system for laser shock peening.

Figure 1a shows the FC-LD-532 laser processing drive control all-in-one machine system from Wuhan Feicheng Optoelectronics Technology Co. (Wuhan, China) This laser quenching experimental equipment system contains a laser, a three-axis moving motor, a water-cooling system, a laser controller, and a programming system. Figure 1b shows the SIA-LP-21 laser shock wave equipment system. The basic parameters of this system are a laser Nd:YAG, pulse width 15 ns, and laser wavelength 1064 nm, and the main components are the laser host, power supply, cooling system, and control system.

### 2.3. Experimental Method

**Figure 2:**
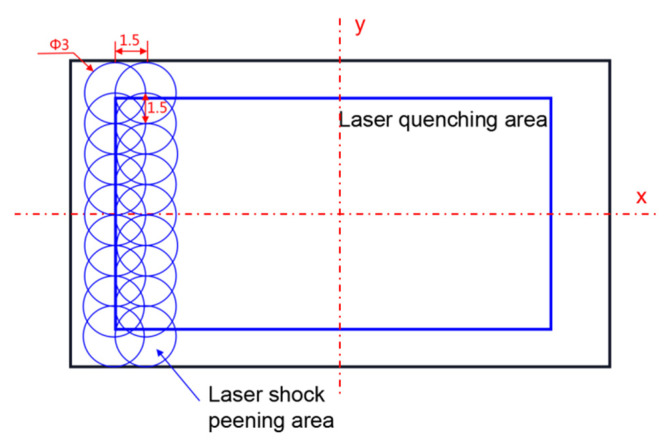
Design of laser quenching–shock peening strengthening experiment scheme.

After the optimization of the preliminary LQ parameters, the surface of the prepared sample was laser quenched and strengthened using the FC-LD-532 laser processing drive control all-in-one machine with a selected power of 1200 w and a scanning speed of 4 mm/s. The surface of the quenched sample was laser shocked using the SIA-LP-21 laser shock wave equipment system, thus achieving LQ-LSP treatment of the sample surface, and the composite strengthening the area is shown in Figure 2.

The laser shock energy was 10 J, spot size 3 mm, lap rate 50%, bound and absorbing layers of water and aluminum foil, respectively, 1–4 impacts; Table 2 shows the experimental control group design.

### 2.4. Representation

Measurement of residual stress values using a residual stress gauge type LXRD from PROTO Canada. For each test, 10 points were selected at different locations on the same plane of the specimen, and the average value was taken as the residual stress value for that surface. The treated Cr12MoV steel was tested for hardness using a Vickers micro hardness tester. The material was analyzed for physical phase using a Bruker D8 ADVANCE X-ray diffractometer. The morphology of the Cr12MoV steel was observed with a Hitachi regulus 8100 field emission scanning electron microscope. The surface micromorphology of the material was observed with an Olympus OLS4100 confocal microscope. Nanoindentation tests were performed on a NanoTest Vantage nanoindenter [19]. The specimens were subjected to wear resistance tests on a CFT-1 friction and wear machine, used to test the wear resistance of the material, with a time setting of 15 min. After the wear test, the surface roughness and the shape of the wear marks were observed by 3D morphometry and SEM.

## 3. Analysis and Conclusions

### 3.1. Surface Morphology and Microstructure Transformation Patterns

**Figure 3:**
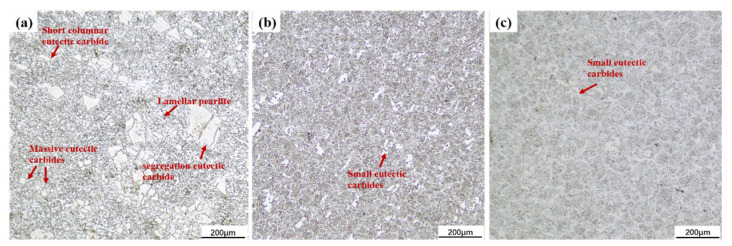
Microstructure of the specimen. (**a**) UT; (**b**) LQ; (**c**) LQ-LSP.

Figure 3a shows that the large reticulated eutectic carbides are scattered on the matrix and the fine carbides are distributed in a fishbone pattern on its left side; the reticulated eutectic carbides are unevenly distributed and over 200 μm in size. The size of the particles was reduced by 50% compared to the UT size. The analysis shows that the LQ temperature reaches the austenite phase transition temperature, and the matrix changes from spherical pearlite to fine martensite. In addition, the LQ specimens had tighter grains, smaller voids between the tissues, and finer eutectic carbides of no more than 80 μm. The LQ-LSP specimens had further grain refinement compared to the LQ specimens in Figure 3b. The analysis suggests that the interaction between the laser shock wave and the material causes the material tissue dislocation and twin splitting, which leads to a further refinement of carbide size with the appearance of the shock wave and a further improvement in the surface properties of the Cr12MoV steel.

**Figure 4:**
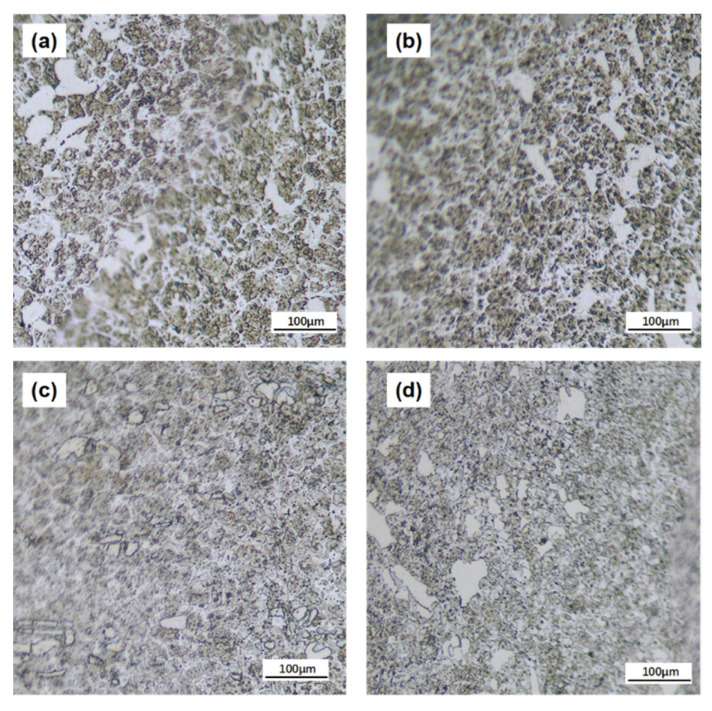
Microstructure of Cr12MoV laser-hardened layer specimens after different numbers of laser shock treatments. (**a**) LQ-LSP1; (**b**) LQ-LSP 2; (**c**) LQ-LSP 3; (**d**) LQ-LSP 4.

In order to investigate the effect of LQ-LSP on the microstructural changes to Cr12MoV steel in more depth, the microstructural changes of Cr12MoV steel under different numbers of laser shocks were therefore investigated. Figure 4a–d shows the microstructure of the laser-hardened layer of Cr12MoV steel after receiving laser shocks 1–4 times, respectively. Combined with the above analysis of the UT and LQ microstructure transformation law, it can be determined that the specimen after LQ still exhibits a large carbide; LQ surface compound LSP can further reduce the size of carbide. As shown in Figure 4, the size of the carbide particles in the specimen decreases as the number of LSP increases. As shown in Figure 4c,d, the carbide particle size of the LQ-LSP 3 specimen was the smallest, with the largest carbide particle size being only 20 μm, which was a significant improvement in size and distribution compared to the LQ-LSP 1 group, and the LQ-LSP 4 carbide particle size increased. The analysis suggests that as the number of LSPs increases, the interaction between the shock wave and the material is strengthened, and the tissue dislocation and twin splitting induced under multiple LSPs has more time and greater energy, leading to further refinement of the carbide size with the increase in the number of impacts and further improvement of the material surface properties. However, at more than three laser shocks, the plastic deformation of the laser-hardened layer surface reaches its limit, and another impact will cause reverse plastic deformation of the material, resulting in an increase in carbide particle size in some areas, thus weakening the strengthening effect of laser shock on the hardened layer of Cr12MoV steel.

**Figure 5:**
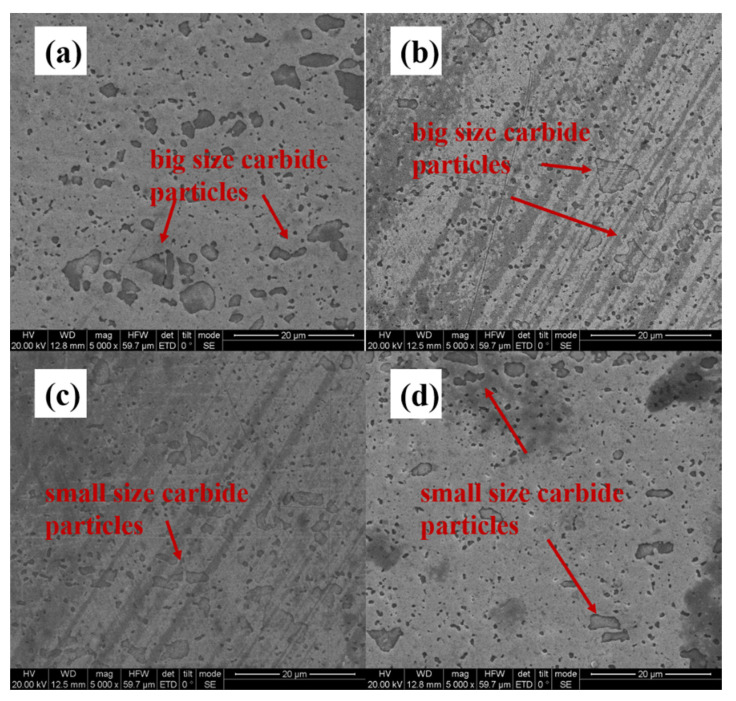
SEM images of samples after different numbers of laser shocks—(**a**) LQ-LSP1; (**b**) LQ-LSP2; (**c**) LQ-LSP3; (**d**) LQ-LSP4.

Figure 5a shows the presence of larger-sized carbides on the surface of the specimen, with a more concentrated distribution. With the increase in the number of LSPs, it can be observed that the grains in Figure 5b,c are gradually refined and more uniformly dispersed. Figure 5c shows that after three laser shocks, the carbide particles on the hardened layer surface are smaller in size and the carbide arrangement is more dispersed. As can be seen from Figure 5d, the plastic deformation of the surface of the LQ-LSP3-hardened layer reaches its limit after three rounds of LSP. Continued impact causes reverse plastic deformation of the material and increases the size of the carbide particles in some areas, thus reducing the strengthening effect of the laser shock peening on the hardened layer [20]. The comparison revealed that LQ-LSP3 has the smallest carbide particles on the surface of the laser-hardened layer and the best strengthening effect.

### 3.2. Analysis of Residual Austenite Content

**Figure 6:**
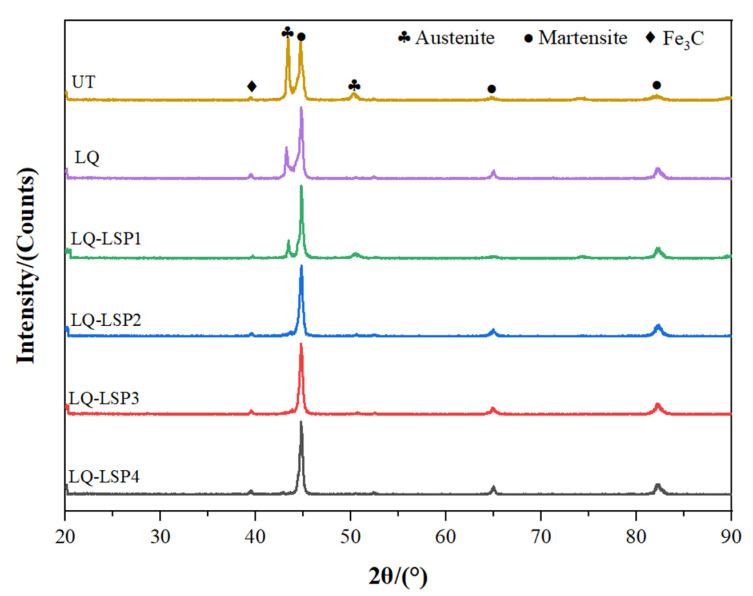
XRD patterns of samples treated by different processes.

Figure 6 shows the XRD diffractograms of the specimens after different process treatments. Neither LQ nor LQ-LSP strengthening produce excess physical phases in Cr12MoV steels. The main physical phases of the material are Fe_3_C, martensite, and residual austenite. LQ and radiation on the surface of the material rapidly heats up and then drops sharply causing the transformation of austenite into martensite. The LQ specimens contain substantially less austenite and more martensite compared to the UT specimens, but they still have more residual austenite present. LQ-LSP specimens were found to have a small reduction in residual austenite content compared to LQ specimens. It is demonstrated that LSP can further transform the residual austenite phase of the laser-hardened layer into martensite.

**Figure 7:**
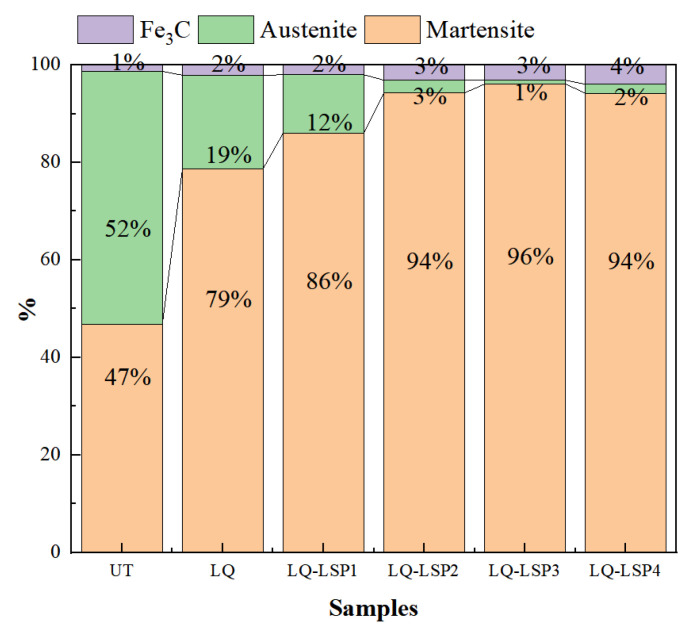
Distribution of phase content of samples after different processes.

As can be seen from Figure 7, the residual austenite content of the hardened layer of the material decreases with the number of impacts; the residual austenite content of the LQ-LSP3 group of steels is only 0.8%, while the martensite content is as high as 96.1%. After LQ, there is still more residual austenite in the organization. This is due, on the one hand, the characteristics of LQ rapid cooling and rapid heating making Cr12MoV steel austenite insufficient, and, on the other hand, martensite strengthening the untransformed austenite, thus preventing the transformation of austenite into martensite and so leaving behind residual austenite. Cr12MoV steel after LQ-LSP enables further conversion of residual austenite into martensite due to the shock wave-induced martensitic phase transformation, which results in a further reduction in residual austenite content [21,22]. After the LQ-LSP, the residual austenite content of the specimen is very low, but it cannot be completely eliminated, indicating that the plastic deformation of the material has reached its limit, and the residual austenite is no longer reduced.

As the required hardness of the surface of the drawing bar needs to be enhanced, the higher the martensite content the better. A comparison shows that LQ-LSP3 has the highest martensite content and the best hardness enhancement.

### 3.3. LQ-LSP Specimen Surface Microhardness Distribution

**Figure 8:**
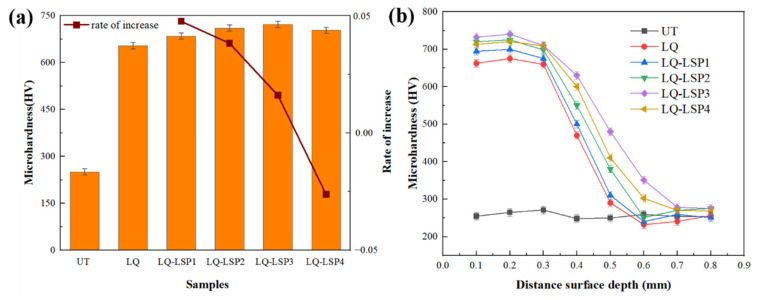
The surface microhardness and cross-sectional hardness distribution of samples treated with different processes—(**a**) surface microhardness; (**b**) cross-sectional hardness.

As can be seen in Figure 8a, the surface microhardness after LQ (653.68 HV) was increased by 160% compared to the UT specimen (250.70 HV). The microhardness of the material increased by 4.8%, 8.6%, 10.5% and 7.6%, respectively, after 1–4 LSP treatments on the basis of laser-hardened specimens. Figure 6 shows that as the number of LSP increases, the content of martensite also increases, and the content of residual austenite decreases. This is due to the fact that a certain temperature is generated at the surface of the LSP which makes some of the residual austenite transform into martensite due to the increase in temperature [23]. LSP on the hardened surface of Cr12MoV steel changes the size of the material surface grains and the martensite content under the radiation of the high-pressure shock wave, thus improving the material’s resistance to plastic deformation and microhardness. The organization of the hardened layer after LQ is fine martensite and residual austenite, which increases the surface hardness. LQ-LSP strengthening promotes more phase transformation of residual austenite into martensite after LQ, and the martensite formed by laser quenching promotes grain refinement under high-pressure shock waves, thereby increasing the surface microhardness of the specimen. The shock wave radiation generated by the LQ is made stronger by the number of impacts that are continuously superimposed. The microhardness increases with the number of impacts, but the growth rate keeps decreasing, and the growth rate decreases to a negative value following a fourth round, indicating that there is an upper limit to the improvements made to the microhardness of the material by LSP. When the material surface reaches the limit of plastic deformation, the impact waves start to amplify from the edge of the area and then gather in the center to produce unloading wave phenomenon on the surface, thus affecting the impact effect [24].

As can be seen in Figure 8b, the maximum hardness induced by laser quenching-shock peening in the depth direction is within 0.2 mm of the specimen surface, with the material hardness gradually decreasing as the depth of the peel increases, eventually converging to the hardness at which it would be without treatment. This is due to the high-pressure shock wave that is generated when the laser acts on the surface of the material. The diffusion of impact energy in the surface layer of the material leads to a gradual weakening process; as the kinetic energy of the shock wave is absorbed and consumed by the material peeling layer and then transformed into deformation energy generated by plastic deformation, the microhardness decreases with the increase of the peeling depth and finally tends to the untreated state [25]. LQ-LSP improves the hardness of Cr12MoV steel in the depth direction by about 0.7–0.8 mm. The microhardness values and depth of influence of the LQ-LSP3 group were higher than those of the remaining three groups, indicating that LQ-LSP3 had the best effect on the microhardness of the material.

### 3.4. Surface Residual Stress Distribution

According to statistics, molds account for 80% of the overall forms of failure due to wear and fatigue failure, of which die stretching bars are one type [26]. Therefore, it is necessary to convert the inherent residual tensile stresses on the surface of the mold Cr12MoV steel into residual compressive stresses, thereby enhancing the fatigue strength of the surface.

**Figure 9:**
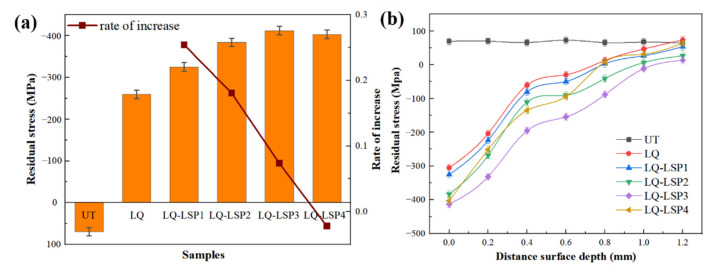
The surface residual stress and section residual stress distribution of samples treated by different processes—(**a**) surface residual stress; (**b**) section residual stress.

As shown in Figure 9a, the residual stress on the surface of the UT material was 69.26 MPa, and the residual compressive stress after LQ increased to −259.29 MPa, an increase of 474.3% in comparison. It can be seen that the LSP can further improve the residual compressive stress on the surface of the laser-hardened layer of the material, and the best number of rounds of LSP is three. The residual compressive stress on the surface increases with the number of impacts, but the growth rate keeps decreasing, with the growth rate decreasing to a negative value by the fourth iteration, due to the fact that the first few LSP have caused severe plastic deformation on the surface, allowing subsequent shock waves to generate deeper plastic deformation and residual compressive stress. When the impact pressure exceeds twice the elastic limit of the material, the material reaches its plastic deformation limit and hardens the surface, thus affecting the growth of residual stresses [27]. LQ and LSP affect the surface residual stresses of the material through martensitic phase transformation and plastic deformation, respectively. Due to the volume expansion triggered by the quenched martensite generated by the LQ, the residual stresses in the quenched layer are not uniformly distributed. The high-pressure shock wave generated by the LSP causes elastic and plastic deformation in the reinforced area, turning the complex residual stresses in the hardened layer into uniform residual compressive stresses. LQ-LSP combines the advantages of both strengthening processes, so that the residual compressive stress of the material is further increased [28,29].

As can be seen in Figure 9b, after LQ-LSP, the residual stress on the surface of the material gradually decreases as the material is delaminated, eventually converging to the magnitude of the stress during UT. This is due to the high-pressure shock wave generated when the laser acts on the surface of the material, and the diffusion of the impact energy on the surface of the material is a gradual weakening process. With the increase in peel depth, the residual compressive stress decreases and finally tends to the untreated state [30]. The depth of the residual stress produced by LQ-LSP strengthened material is about 1–1.2 mm, and the residual compressive stress of the LQ-LSP3 group is the largest and the depth of influence deepest, indicating that the laser-hardened layer combined with LQ-LSP3 has the best effect on the residual compressive stress of the material.

### 3.5. Nanoindentation Load–Depth Curves, Nanohardness, and Elastic Modulus Influence Laws

**Figure 10:**
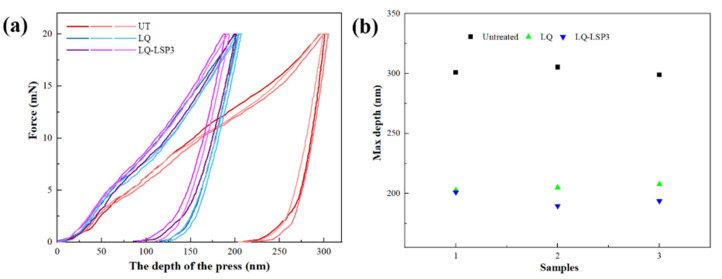
Nanoindentation load–depth curves and the maximum nanoindentation depth of specimens after different processes. (**a**) Nanoindentation load–depth curves; (**b**) maximum nanoindentation depth.

Figure 10a shows the nanoindentation load–depth curves and the maximum nanoindentation depth of the specimens after different processes. LQ-LSP3 is a sample that has undergone three laser shocks after LQ. As can be seen from the test curves of the three groups of specimens, the curves of the UT specimens are more to the right than the other two groups, and there are larger displacements of their maximum load depth and unload depth. The curves of the LQ and LQ-LSP specimens are more similar, but the curve of the LQ-LSP specimen is more to the left, indicating that less slippage occurs on the surface of the LQ-LSP3 specimen when subjected to loading. Figure 10b shows the maximum nanoindentation depth produced on the surface when a 20 mN load is applied to the specimen using a diamond Bosch indenter. The average depths of the three groups of specimens were 301.69 nm, 205.12 nm, and 194.77 nm, respectively. The LQ specimens showed less slip than the UT specimens under load, because the LQ induced martensite generation on the material surface, which resulted in a refinement of the tissue grains and a more significant improvement to the plastic deformation resistance of the material. The LQ-LSP specimens were loaded with the least slip, indicating that the LQ-LSP3 can further refine the organization of the laser-hardened layer and obtain better resistance to plastic deformation.

**Figure 11:**
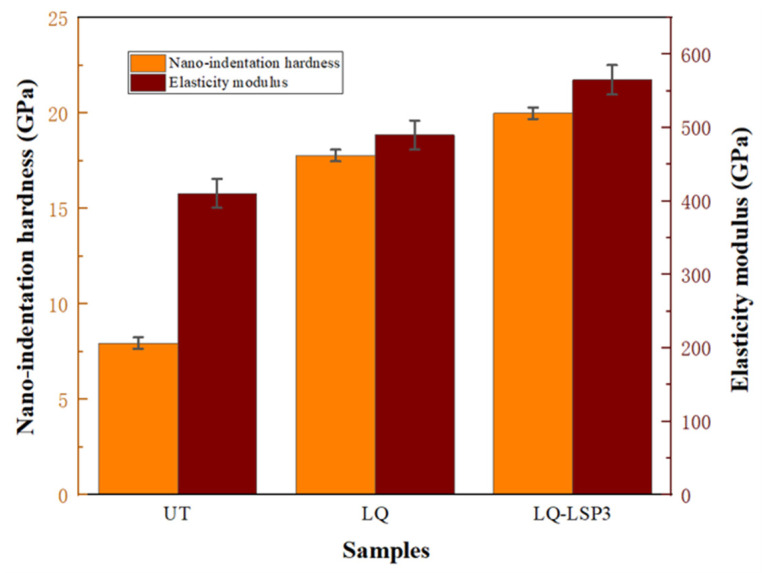
Nanohardness and elastic modulus of samples treated by different processes.

The indentation data are analyzed and integrated to obtain the nanohardness and the approximate modulus, which can be calculated using the material Poisson’s ratio (0.28 for Cr12MoV) in order to obtain the material’s modulus of elasticity. Figure 11 shows the elastic modulus and nanohardness of different specimens, and the parameters of the material after LQ are also more excellent compared to the UT specimens. The surface nanohardness and elastic modulus parameters of the specimens after LQ-LSP were the most excellent and improved by 12.4% and 15.3%, respectively, compared to the LQ specimens. This indicates that the LSP3 technique can further improve the surface properties of laser-hardened layers. LQ causes the austenite phase transformation on the surface of Cr12MoV steel to generate fine martensite, which refines the grains and therefore increases the hardness and plastic deformation resistance of the material, resulting in an increase in the nanohardness and elastic modulus of the LQ specimens. The high-energy shock wave of LSP3 acts on the hardened layer, promoting further refinement of tissue grains, grain refinement leads to more grain boundaries, making it more difficult for the material to deform plastically, thus improving the material’s ability to resist plastic deformation and improving the nanohardness and elastic modulus [31].

### 3.6. Surface Wear Resistance Research

**Figure 12:**
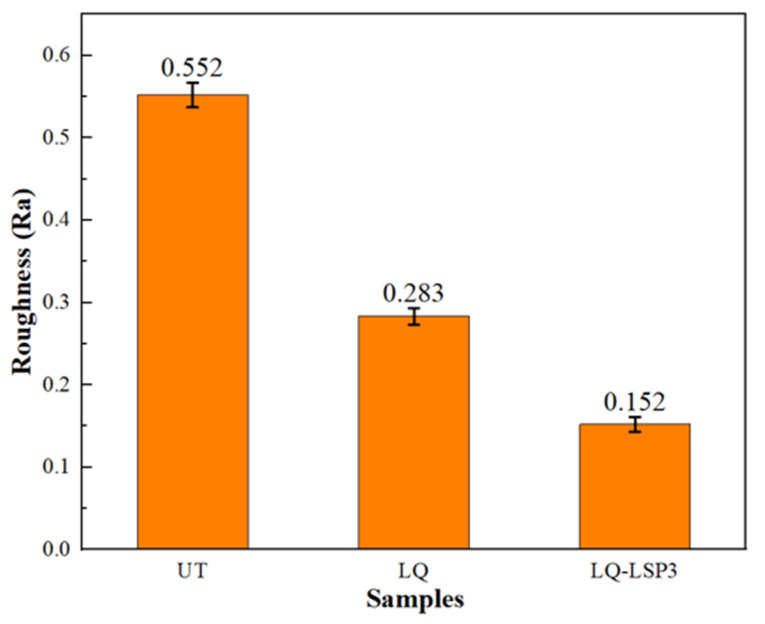
Roughness in the area of wear scratches for different specimens.

The roughness of the wear specimen surface reflects the degree of wear on the specimen. The smaller the roughness, the flatter the surface, and the fewer the spalling marks, the better the wear resistance will be. As can be seen from Figure 12, the roughness of Cr12MoV steel after LQ treatment was reduced by 48.7% as compared to the UT specimen, indicating that the LQ process can effectively improve the surface roughness of the material. The roughness of Cr12MoV steel after LQ-LSP3 was reduced by 72.5% as compared to the UT specimen, and the LQ was more effective than FQ in improving the wear resistance of Cr12MoV steel, with the LQ-LSP3 strengthening being the best in terms of improving the wear resistance of the material. Hardness is an important basis for good wear resistance in mold steels. Combined with the Holm–Archard wear law [32] and the wear damage mechanism [33], in plunge wear, the greater the surface hardness of the material, the better its wear resistance, and the less the wear affects the surface finish of the material, and the less its surface roughness. LQ is achieved by turning the martensitic phase into fine martensite, which improves the material surface hardness and wear resistance [34] and reduces the roughness of the wear surface. The wear surface roughness after LQ-LSP3 strengthening is the smallest, mainly because LQ-LSP3 promotes plastic deformation, grain refinement, and improved microstructure morphology and distribution of the material, which leads to the hardness and wear resistance of the material [35].

**Figure 13:**
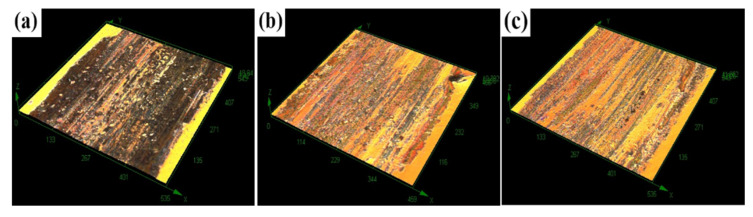
The 3D morphology of the wear area of samples treated by different processes—(**a**) UT; (**b**) LQ; (**c**) LQ-LSP3.

Figure 13 shows the morphological analysis of the abrasion marks of the specimens treated with different processes. The surface of the UT specimen, as shown in Figure 13a, is severely worn and has a large number of scratches and pits, and a large amount of black accumulation is gathered along both sides of the wear direction of the abrasion marks, increasing with the increase of the depth of the scratches, thereby indicating that the damage to the UT specimen is greater. Figure 13b shows that the surface plastic flow traces, craters, and scratch sizes of the specimens were reduced to different degrees after the LQ treatment, the overall wear level was reduced, and the frictional wear was improved. Observing the morphology of the abrasion marks after LQ-LSP3, as shown in Figure 13c, it is found that the scratches of the specimens after laser composite strengthening are very shallow and mainly dominated by plastic removal. The specimen surface did not have a large number of pits or black accumulation caused by scratch damage, and the surface wear marks tended to be flat. This indicates that the wear resistance of Cr12MoV steel has been significantly improved after LQ-LSP3 strengthening.

**Figure 14:**
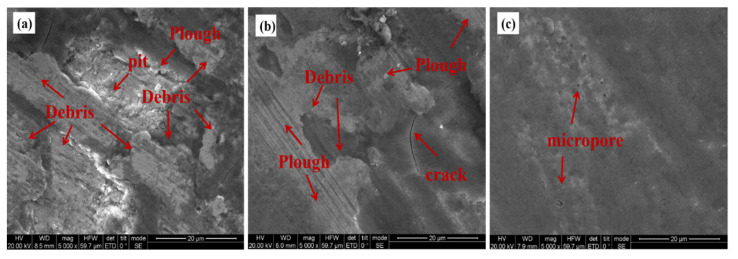
SEM morphologies of the wear marks of samples treated by different processes—(**a**) UT; (**b**) LQ; (**c**) LQ-LSP3.

Figure 14 shows the SEM morphology of the abrasion marks of different specimens. Figure 14a shows that the surface of the UT specimen is severely damaged with a significant height difference and a high distribution of bonded debris, deep pits, cracks, and gouges on the abrasion surface. Figure 14b shows the narrowing and shallowing of the plough grooves on the surface of the specimen after LQ with no obvious pits and only a few micro-cracks, indicating that the LQ process can effectively improve the wear resistance of the material. As shown in Figure 14c, the wear surface of the LQ-LSP3-reinforced specimens showed no significant debris spalling marks compared with the other two groups, and the overall surface was the flattest and cleanest, with only a few shallow furrows and micro-porosity, which showed slight adhesive wear and abrasive wear. This indicates that the LQ-LSP3 strengthening has further improved the wear resistance of the material.

**Figure 15:**
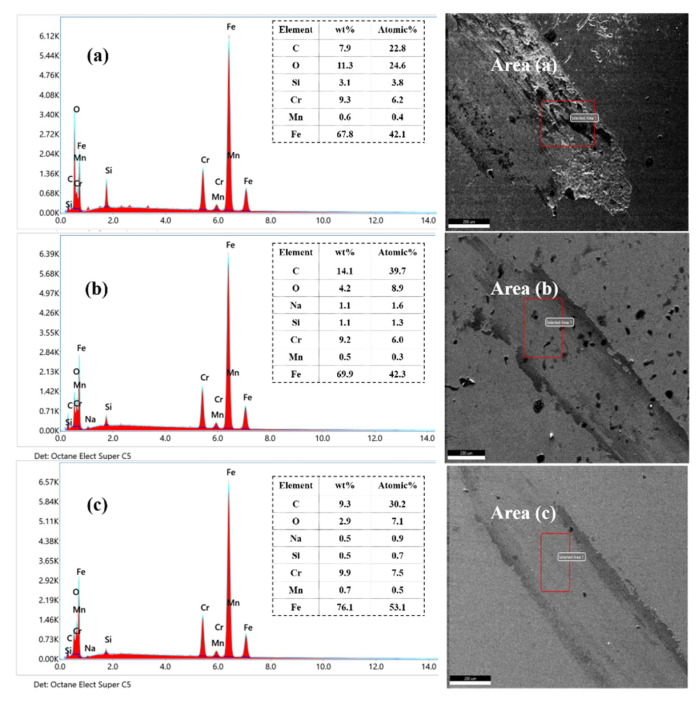
EDS spectra of samples treated by different processes—(**a**) UT; (**b**) LQ; (**c**) LQ-LSP3.

As shown in Figure 15, the oxygen content of the four samples is 24.6%, 8.9%, and 7.1%, respectively, indicating that the oxidation wear of Cr12MoV steel is reduced after LQ-LSP3. The base surface of UT specimens is spalling due to frictional wear, forming more pits and cracks. Oxide abrasives are more likely to adhere and accumulate on rough surfaces, and more oxidative wear will occur when the severely damaged surface is exposed to more oxygen in the air, resulting in more oxygen in the specimen [36]. LQ and LQ-LSP3 specimens have less exfoliation and oxidation, and the surface morphology remains basically flat and less prone to the adhesion of oxidation and severe oxidative wear.

**Figure 16:**
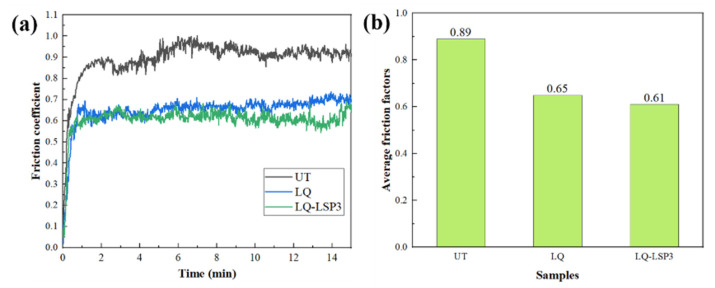
(**a**) Friction coefficient; (**b**) average friction factors.

Figure 16a shows the friction coefficient curves of three sets of wear specimens. The value of the friction coefficient is small in the initial stage of the wear test and ends in a rapid rising stage within 1 min of the wear test. In the early stages of wear, the specimens are in the abrasive wear phase, which is mainly characterized by adhesive wear and small values of the coefficient of friction. As the wear test proceeds, the metal abrasive chips generated by the mutual grinding of the specimen and the friction sub are involved in the contact surface of the two friction subs, and the adhesive wear gradually turns into abrasive wear, resulting in a rapid increase in the coefficient of friction [37]. The three sets of specimens entered a stable wear phase within 2 min, with the amplitude of the friction curve gradually levelling off. From Figure 16b, it can be seen that the average friction coefficients of the three groups of specimens are 0.89, 0.65, and 0.61, and the average friction coefficients of the group specimens are reduced by 27% and 32%, respectively, compared to the UT specimens. This analysis leads to the conclusion that the smaller the coefficient of friction, the better the wear resistance of the LQ-LSP3 specimens from the principle of [38].

**Figure 17:**
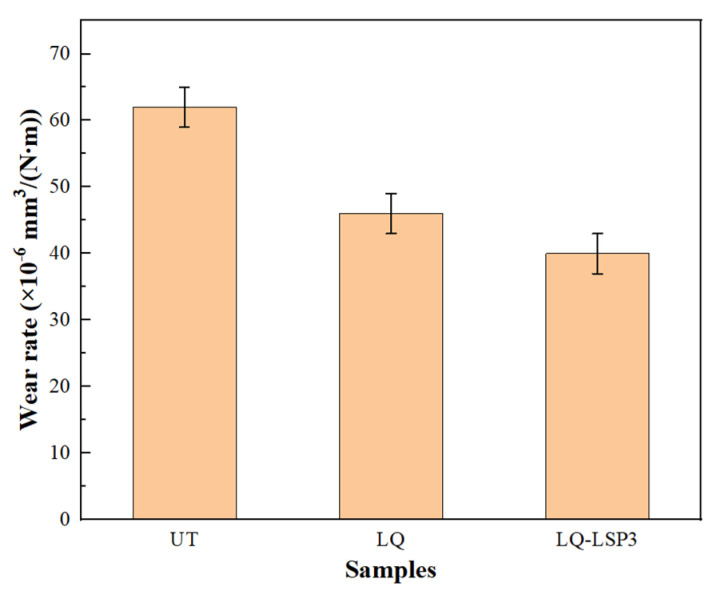
Wear rate of specimens treated with different processes.

Figure 17 shows the wear rates of the specimens treated by the different processes; these rates were calculated from the wear rate equation by analyzing the 3D morphology of Figure 13 using the MFT software and measuring the wear volume on the surface of the specimens. The wear rates for the three sets of specimens were 62 × $10^{-6}{\mathrm{mm}}^{3}/\left(N\cdot m\right),\;$46 × $10^{-6}{\mathrm{mm}}^{3}/\left(N\cdot m\right)$ and 40 × $10^{-6}{\mathrm{mm}}^{3}/\left(N\cdot m\right)$, respectively, with the LQ and LQ-LSP3 specimens showing a 25.81% and 35.48% reduction in wear rate, respectively, compared to the UT specimens. The friction coefficient, wear rate, and microstructural transformation are closely related, with the increased martensite content of LQ and LQ-LSP3 compared to UT resulting in increased hardness, reduced friction coefficient, reduced number of surface scratches, and shallower pear grooves. These phenomena also demonstrate the reduced wear rate of the LQ and LQ-LSP3 specimens. The analysis shows that the LQ-LSP3 specimen has the lowest wear rate, the least surface scratches, and the shallowest pear grooves, further demonstrating that LQ-LSP3 has the most significant improvement in the wear resistance of Cr12MoV steel.

## 4. Conclusions

A study of the microstructure and mechanical properties of Cr12MoV steel by LQ-LSP strengthening was carried out in order to investigate the differentiated forms of failure of the dangerous end faces of the tension bars. The main conclusions are as follows.

(1) The microstructure of the material was observed by SEM and metallurgical microscopy, and the results showed that the LQ tissue was mainly martensite and residual austenite. Compared with the pearlite of UT specimens, the density and organization of martensite are small, and the hardness is high; the microstructure of the LQ-LSP3 group specimens has the smallest carbide particle size. The largest carbide particle size is only 10 μm, and the carbide distribution is the most dispersed, with the smallest carbide density per unit area. This indicates that LQ-LSP3 can effectively improve the surface properties of Cr12MoV.
(2) The observation of the physical phase of Cr12MoV steel by X-ray diffractometer shows that LQ-LSP strengthening can effectively reduce the residual austenite content of the material and does not produce other phases, the main phase of which is Fe_3_C. After the LQ treatment of Cr12MoV steel, the residual austenite content was reduced from 51.6 to 19.2%, with the lowest residual austenite content at 0.8% and a high martensite content of 96.1% in LQ-LSP3 specimens.
(3) A study of the microstructure and mechanical properties of Cr12MoV steel by LQ-LSP strengthening was carried out in order to address the differentiated forms of failure of the dangerous end faces of the tension bars. The study showed that the specimens in the LQ-LSP3 group had the highest surface microhardness of 722.30 HV, an increase of 288.1% compared to the untreated specimens and an increase of 10.5% in microhardness compared to the LQ specimens. It also induced the largest residual pressure amplitude of −412.08 MPa, an increase of 690.5% compared to the untreated specimens and 59.0% compared to the LQ specimens in terms of residual compressive stress.
(4) A study of the resistance to plastic deformation and wear resistance of LQ-LSP-strengthened Cr12MoV steel showed that the specimens strengthened by LQ-LSP had the smallest displacement of the nano-load–depth curve. This exhibits the greatest nanohardness (20.0 Pa) and modulus of elasticity (565.25 Pa), while reducing the friction coefficient (0.61), surface roughness (0.152 Ra), and wear rate (40 $\times 10^{-6}{\mathrm{mm}}^{3}/\left(N\cdot m\right)$). The smooth and flat surface of the specimen, with shallow and narrow plough grooves, improves the resistance of Cr12MoV steel to plastic deformation and wear.

## Figures and Tables

**Table 1:** Cr12MoV steel chemical composition.

C	Si	Mn	S	P	Cr	Ni	Cu	V	Mo	Fe
1.45–1.70	≤0.40	≤0.40	≤0.03	≤0.03	11–12.50	≤0.25	≤0.30	0.9–1.40	0.15–0.50	Bal.

**Table 2:** Control group design of Cr12MoV laser quenching–shock peening composite strengthening experiment.

Samples	Treatment
UT	Untreated
LQ	Laser Quenching (1200 W, 4 mm/s)
LQ-LSP1	Laser Quenching (1200 W, 4 mm/s) after Once Laser Shock Peening (10 J)
LQ-LSP2	Laser Quenching (1200 W, 4 mm/s) after twice Laser Shock Peening (10 J)
LQ-LSP3	Laser Quenching (1200 W, 4 mm/s) after Thrice Laser Shock Peening (10 J)
LQ-LSP4	Laser Quenching (1200 W, 4 mm/s) after Quartic Laser Shock Peening (10 J)

## Data Availability

The data that support the findings of this study are available from the corresponding author upon reasonable request.

## References

[1] Xu Y., Du Z., Ruan L., Zhang W. (2016). Research status and development of laser shock peening. J. Laser Appl..

[2] Sun G., Li G., Gong Z., Cui X., Yang X., Li Q. (2010). Multiobjective robust optimization method for drawbead design in sheet metal forming. Mater. Des..

[3] Yu H. (2004). Development of drawbead investigation in sheet metal forming technology. J. Plast. Eng..

[4] Trzepiecinski T., Fejkiel R. (2019). A 3D FEM-Based Numerical Analysis of the Sheet Metal Strip Flowing Through Drawbead Simulator. Metals.

[5] Trzepiecinski T., Kubit A., Slota J., Fejkiel R. (2019). An Experimental Study of the Frictional Properties of Steel Sheets Using the Drawbead Simulator Test. Materials.

[6] Kong D.J., Xie C.Y. (2015). Effect of laser quenching on fatigue properties and fracture morphologies of boronized layer on Cr12MoV steel. Int. J. Fatigue.

[7] Roshchupkin V.V., Lyakhovitskii M.M., Pokrasin M.A., Minina N.A., Kudryavtsev E.M. (2019). Effect of Quenching on the Microhardness of Steels. Russ. Metall..

[8] Liu Y., Tian Y., Zhang H., Zhang H., Li Y. (2019). Microstructure and properties of Cr12MoV die steel by laser quenching with different power. IOP Conf. Ser. Mater. Sci. Eng..

[9] Huangliang S., Jiqiang L., Jiazhi X., Shi H.L., Li J.Q., Jia Z.X., Liu L.Z. (2014). Research on Properties of Cr12 Steel Processed by Laser-Remelting. Laser Optoelectron. Prog..

[10] Shen X., Shukla P., Subramaniyan A.K., Zammit A., Swanson P., Lawrence J., Fitzpatrick M.E. (2020). Residual stresses induced by laser shock peening in orthopaedic Ti-6Al-7Nb alloy. Opt. Laser Technol..

[11] Zhang C., Dong Y., Ye C. (2021). Recent developments and novel applications of laser shock peening: A review. Adv. Eng. Mater..

[12] Lu Y., Zhao J., Qiao H., Hu T., Sun B., Wu J. (2019). A study on the surface morphology evolution of the GH4619 using warm laser shock peening. AIP Adv..

[13] Guan L., Ye Z., Zhong J., Li Y., Zhang Y. (2022). Enhancement of corrosion resistance of 304L stainless steel treated by massive laser shock peening. Opt. Laser Technol..

[14] Ge M.Z., Tang Y., Zhang Y.K., Wang Y. (2022). Enhancement in fatigue property of Ti-6Al-4V alloy remanufactured by combined laser cladding and laser shock peening processes. Surf. Coat. Technol..

[15] Hepeng Z. (2022). Laser Shock Peening Induced Surface Gradient Structural Evolution and Crack Growth Characteristics of TC4 Titanium Alloy.

[16] Hu X., Zhao J., Teng X., Nie X., Jiang Y., Zhang Y. (2022). Fatigue Resistance Improvement on Double-Sided Welded Joints of a Titanium Alloy Treated by Laser Shock Peening. J. Mater. Eng. Perform..

[17] Ranjith Kumar G., Rajyalakshmi G., Swaroop S., Arul Xavier Stango S., Vijayalakshmi U. (2019). Laser shock peening wavelength conditions for enhancing corrosion behaviour of titanium alloy in chloride environment. J. Braz. Soc. Mech. Sci. Eng..

[18] Shaoxiang Q. (2021). Research on Microstructure, Strengthening Mechanism and Performance of Laser Cladding Nickel-Based Alloy and Its Surface Laser Peening.

[19] Yan W.Z., Li Y.L., Wen Z.X. (2020). Effect of crystallographic orientation on nano-indentation behavior of nickel based single crystal super alloys. Rare Met. Mater. Eng..

[20] Fairand B.P., Wilcox B.A., Gallagher W.J., Williams D.N. (1972). Laser shock-induced microstructural and mechanical property changes in 7075 aluminum. J. Appl. Phys..

[21] Ren X.D., Zhang Y.K., Yongzhuo H.F., Ruan L., Jiang D.W., Zhang T., Chen K.M. (2011). Effect of laser shock processing on the fatigue crack initiation and propagation of 7050-T7451 aluminum alloy. Mater. Sci. Eng. A.

[22] De Diego-Calderón I., Rodriguez-Calvillo P., Lara A., Molina-Aldareguia J.M., Petrov R.H., De Knijf D., Sabirov I. (2015). Effect of microstructure on fatigue behavior of advanced high strength steels produced by quenching and partitioning and the role of retained austenite. Mater. Sci. Eng. A.

[23] Tong C., Tantan H., Sanming D., Yang L., Jingkai L., Yang L., Yongzhen Z. (2022). Effect of Laser Shock Processing on Microstructure and Tribological Behavior of GCr15 Bearing Steel. Surf. Technol..

[24] Liu Q., Yang C.H., Ding K., Barter S.A., Ye L. (2007). The effect of laser power density on the fatigue life of laser-shock-peened 7050 aluminium alloy. Fatigue Fract. Eng. Mater. Struct..

[25] Wang J.T., Zhang Y.K., Chen J.F., Zhou J.Y., Luo K.Y., Tan W.S., Sun L.Y., Lu Y.L. (2017). Effect of laser shock peening on the high-temperature fatigue performance of 7075 aluminum alloy. Mater. Sci. Eng. A.

[26] Jingui L. (2002). Surface Hardening Technologies and Mould Service Life. China Surf. Eng..

[27] Ballard P. (1991). Residual Stressed Induced by Rapid Impact-Applications of Laser Shocking. Ph.D. Thesis.

[28] Kattoura M., Mannava S.R., Dong Q., Vasudevan V.K. (2017). Effect of Laser Shock Peening on Elevated Temperature Residual Stress, Microstructure and Fatigue Behavior of ATI 718Plus Alloy. Int. J. Fatigue.

[29] Lu J.Z., Luo K.Y., Zhang Y.K., Cui C.Y., Sun G.F., Zhou J.Z., Zhang L., You J., Chen K.M., Zhong J.W. (2010). Grain refinement of LY2 aluminum alloy induced by ultra-high plastic strain during multiple laser shock processing impacts. Acta Mater..

[30] Babu P.D., Marimuthu P. (2019). Status of laser transformation hardening of steel and its alloys: A review. Emerg. Mater. Res..

[31] Sherif H.A., Almufadi F.A. (2018). Analysis of elastic and plastic impact models. Wear.

[32] Koslowski M. (2010). Effect of grain size distribution on plastic strain recovery. Phys. Rev. B.

[33] Jankauskas V., Skirkus R. (2013). Steel abrasive wear forecasting by wearing surfaces microgeometric parameters/Plienu abrazyvinio dilimo prognozavimas pagal dylancio pavirsiaus mikrogeometrinius parametrus. Mechanics.

[34] Hua W. (2019). Comparative Study on Wear Properties and Wear Mechanism of Austenite and Martensite Wear-Resistant Steel. Shanxi Coking Coal Sci. Technol..

[35] Qiao X., Cheng A., Nie X., Ning M. (2018). A study on die wear prediction for automobile panels stamping based on dynamic model. Int. J. Adv. Manuf. Technol..

[36] Corrochano J., Walker J.C., Lieblich M., Ibáñez J., Rainforth W.M. (2011). Dry sliding wear behaviour of powder metallurgy Al–Mg–Si alloy-MoSi_2_ composites and the relationship with the microstructure. Wear.

[37] Trevisiol C., Jourani A., Bouvier S. (2017). Effect of martensite volume fraction and abrasive particles size on friction and wear behaviour of a low alloy steel. Tribol. Int..

[38] Hui D., Pengfei G., Long X., Kang K.X. (2022). Effect of Heat Treatment Temperature on Microstructure and Friction and Wear Properties of High-speed Laser Cladded Ni/316L Coating. Surf. Technol..

